# The Zebrafish Perivitelline Fluid Provides Maternally-Inherited Defensive Immunity

**DOI:** 10.3390/biom10091274

**Published:** 2020-09-03

**Authors:** Javiera F. De la Paz, Consuelo Anguita-Salinas, César Díaz-Celis, Francisco P. Chávez, Miguel L. Allende

**Affiliations:** 1 FONDAP Center for Genome Regulation, Faculty of Sciences, University of Chile, RM 7800003 Santiago, Chile; javiera.delapaz@gmail.com (J.F.D.l.P.); c.anguitasalinas@gmail.com (C.A.-S.); 2 Laboratory of Systems Microbiology, Faculty of Sciences, University of Chile, RM 7800003 Santiago, Chile; cesardiazcelis@gmail.com; 3 Danio Biotechnologies SpA, RM 7800003 Santiago, Chile

**Keywords:** innate immunity, alpha-2-Macroglobulin, maternal immunity, lectin, chitinase, proteomics

## Abstract

In the teleost egg, the embryo is immersed in an extraembryonic fluid that fills the space between the embryo and the chorion and partially isolates it from the external environment, called the perivitelline fluid (PVF). The exact composition of the PVF remains unknown in vertebrate animals. The PVF allows the embryo to avoid dehydration, to maintain a safe osmotic balance and provides mechanical protection; however, its potential defensive properties against bacterial pathogens has not been reported. In this work, we determined the global proteomic profile of PVF in zebrafish eggs and embryos, and the maternal or zygotic origin of the identified proteins was studied. In silico analysis of PVF protein composition revealed an enrichment of protein classes associated with non-specific humoral innate immunity. We found lectins, protease inhibitors, transferrin, and glucosidases present from early embryogenesis until hatching. Finally, in vitro and in vivo experiments done with this fluid demonstrated that the PVF possessed a strong agglutinating capacity on bacterial cells and protected the embryos when challenged with the pathogenic bacteria *Edwardsiella tarda.* Our results suggest that the PVF is a primitive inherited immune extraembryonic system that protects the embryos from external biological threats prior to hatching.

## 1. Introduction

During oocyte maturation in animals, diverse molecular traits are acquired maternally. These elements are essential for proper embryonic development and for surviving different types of environmental threats. In the case of animals with external fertilization and development, as is the case of most teleost fishes and other aquatic vertebrate and invertebrate animals, the vertical transfer of defensive molecules is critical, as they protect the free-living embryos and larvae before the development and maturation of the immune system. In zebrafish, the presence of adaptive and innate immune system molecules with antimicrobial activity has been reported in the egg cytosol. Proteins such as antibodies [1], complement system proteins [2], and lysozymes [3] support the hypothesis of maternal inheritance of defensive molecules in fish. In teleost eggs, the embryo itself is embedded within the perivitelline fluid (PVF), an extraembryonic fluid that fills the perivitelline space between the embryo and the chorion and partially isolates it from the external environment.

In zebrafish, the PVF is formed during egg activation. The cortical reaction entails a massive exocytosis of the cortical granules present in the periphery of the egg cell. Numerous macromolecules, including glycoproteins, mucopolysaccharides, and lectins, are released into the space between the vitelline membrane and the cell membrane of the egg. This reaction results in the entry of water into the perivitelline space and the consequent expansion of the chorion and formation of the PVF [4,5,6], which likely contains most of the proteins released from the cortical granules. Nonetheless, other embryonic proteins may also enter this fluid at later stages, an aspect that has not been studied. The ultrastructure and exocytosis mechanism of cortical granules has been characterized in mammals, amphibians, crustaceans, and echinoderms; their molecular content has been only partially reported for some vertebrates [7], although it has been well characterized for the apple snail (*Pomacea caniculata*) [8]. In any case, the exact content of cortical granules or the PVF in zebrafish—or any other vertebrate—have not been reported, and therefore the molecular composition of the PVF remains unknown in vertebrates. Both extraembryonic elements, the chorion and the PVF, protect the embryo from dehydration and mechanical injury throughout embryogenesis until hatching, which occurs two days after fertilization. Specifically, the PVF provides mechanical protection and maintains osmotic balance; however, a role for PVF as a defensive barrier against bacterial pathogens has not been reported.

In this work, we first aimed to characterize the global proteomic profile of the PVF in zebrafish during embryogenesis in order to search for molecules that could potentially be involved in inherited immune defense. Secondly, we set out to test whether the PVF possesses defensive properties by carrying out in vitro assays using gram negative bacteria, and in vivo experiments by challenging zebrafish embryos with bacterial infection. A summary of the workflow to establish the defensive function of the PVF is presented in Figure S1.

## 2. Materials and Methods

### 2.1. Workflow

In vitro and in vivo approaches were used to characterize the PVF composition, its direct effect on bacterial cells, and its protection of the zebrafish embryo against biological and physical threats.

### 2.2. Animals

Zebrafish (*Danio rerio*) were maintained and raised in our facility according to standard procedures [9] and uniform controlled parameters, which reduced the variability among individuals. Adult zebrafish were raised using a 14 h light/10 h dark cycle and fed once a day with Gemma 500 formula (Skretting). The embryos were collected by natural spawning from wild type fish of different strains (TAB5, AB, and WT), and batches were raised at 28 °C in E3 medium (5 mM NaCl, 0.17 mM KCl, 0.33 mM CaCl_2_, 0.33 mM MgSO_4_, equilibrated to pH 7.0) in Petri dishes until PVF extraction or treatment. Approximately 300 embryos were used of each stage for proteomic analysis. We estimate that each embryo contained about 0.1–0.3 µL of PVF. All of the procedures complied with the guidelines and were approved by the Institutional Animal Care and Use Committee of the University of Chile (CICUA Certificate #18141-FCS-UCH), considering that the number of animals used was the minimum required for proper statistical validation of the experiments and that animal welfare was ensured.

### 2.3. Peptide Sequencing, Identification, Function, and Origin

The PVF was collected from embryos at 0–2 hpf, 1 dpf, and 2 dpf using clean and disinfected fine forceps to cut the chorions and allow the release of the PVF on a smooth surface to collect the fluid with a micropipette (see Figure S2). Only 0–2 hpf embryos were fixed in 4% paraformaldehyde before PVF extraction to avoid damage to the blastoderm and sample contamination. The 1 dpf and 2 dpf embryos were alive at the moment of extraction. Samples with 100 µL of pure PVF extracted from each stage (approximately 150 µg total protein) were processed for protein extraction and sent for peptide sequencing by Shotgun proteomics, followed by tandem mass spectrometry (MS/MS) in a Q-Exactive HF-X Orbitrap mass spectrometer (Bioproximity LLC, Manassas, VA, USA). This analysis was performed twice with samples from embryos obtained from different batches of fish. The UNIPROT (Universal Protein Resource) identification code (ID) for most of the protein sequences was obtained. These IDs were then searched in several databases (ZFIN, ENSEMBL) to obtain the identity and function of each protein; when no positive results were obtained, a BLASTp (Basic Local Alignment Search Tool for proteins) analysis was performed to predict a protein family and function based on conserved domains, using the Conserved Domain tool at the NCBI website. Further functional prediction and analysis of statistical overrepresentation of protein classes based on Fisher’s test was done using the PANTHER (Protein ANalysis THrough Evolutionary Relationships, http://www.pantherdb.org/) classification system, version 13.1 (Released on 2 March 2018). The PANTHER Overrepresentation Test (Released 12 May 2017) compares the number of protein classes according to Gene Ontology (GO) that are significantly higher that the number of proteins that can be expected to be expressed by chance, in relation with their representation in the whole zebrafish genome (genes in database); results are presented as fold enrichment [10,11,12]. 

Finally, the maternal or zygotic origin of each protein present in the PVF was suggested by their presence or absence in the PVF sampled before the mid-blastula transition (MBT; 0–2 hpf) and the information cross-referenced with an RNAseq study made by Harvey et al. (2013) [13].

### 2.4. Bacterial Strains and Culture Conditions

*Escherichia coli* (DH5α) and *Salmonella typhimurium* (ΔaroA) transformed with the pDiGc vector (#59322 Addgene) to obtain GFP (green fluorescent protein) constitutive expression, were cultured on LB (Luria-Bertani) medium with ampicillin (100 μg/mL) at 37 °C. *Edwardsiella tarda* (FL60) was grown on TSB (tryptic soy broth) with tetracycline (15 μg/mL) at 28 °C.

### 2.5. Bacterial Growth Inhibition Test

Soft agar plates were prepared adding 4 mL of 0.6% (*p/v*) LB-agar mixed with 300 µL of overnight bacterial cultures, on top of hard LB-agar (1.5% *p/v*). Once the plates were solidified, drops of 15 µL and 30 µL of PVF were added, and the plates were incubated at 37 or 31 °C. The appearance of an inhibition halo was evaluated after 4 and 12 h. Phosphate saline buffer (PBS) and gentamicin were used as negative and positive controls, respectively.

### 2.6. Bacterial Agglutination Assay

Live *E. coli* and *S. typhimurium* bacteria expressing GFP were used to detect bacterial clumps as an indicator of an agglutination reaction. Briefly, approximately 10^6^ bacteria from overnight cultures were resuspended in 40 µL of PVF, or PBS as a negative control, and incubated for three hours at 28 °C and for an additional twelve hours at 4 °C, to reduce cellular movement. Finally, 8 µL of these suspensions were dropped on clean slides for their direct observation on a Carl Zeiss LSM 510-META confocal microscope with a 63× objective.

### 2.7. Serological Testing

*E. tarda* and *E. coli* overnight cultures were diluted to reach an optical density equal to 0.5 at 600 nm. Then, 40 µL samples of serial dilutions with PVF or PBS were incubated for 4 h in a 96 well plate with curved bottom wells (96U), and the agglutination reaction was evaluated at 0.5, 1, 3, and 4 h. The formation of a bacterial mat on the bottom of the well is an indicator of a positive agglutination reaction.

### 2.8. Bacterial Infection Challenge

Five to eight groups of three dechorionated embryos (1 dpf and 2 dpf) were incubated in a 96U plate for four hours at 28 °C in 40 µL of bacterial suspension of *E. tarda* (OD 600nm: 0.3; exponential growth phase), resuspended in PBS (positive control of infection) or 50% PVF (treatment). Exposure to PBS in the absence of the pathogen was used as a negative control. After the incubation time, the embryos were washed at least five times with fresh E3 medium and transferred to a new 96U plate. Embryo incubation proceeded for three days when the incubation was started at 1 dpf, and for four days when the exposure was begun at 2 dpf. The final volume of incubation after the bacterial challenge was 150 µL of E3 in each well. The E3 medium was renewed, and survival was monitored daily.

## 3. Results and Discussion

**Figure 1:**
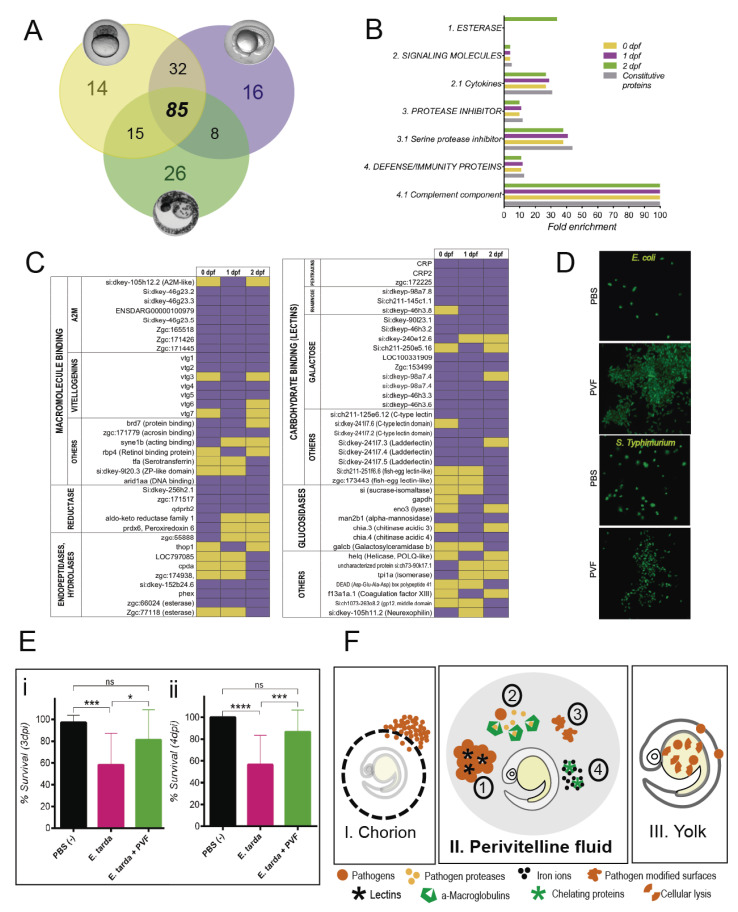
(**A**) The Venn diagram shows the number of exclusive and shared proteins present in the perivitelline fluid (PVF) at different stages: early blastula (yellow), at 1 dpf (purple) and 2 dpf (green). The 85 sequences present at all developmental stages are considered as constitutive of PVF composition. (**B**) Enriched protein classes (according to their Gene Ontology) detected in the PVF. The overrepresentation analysis reveals a marked overrepresentation of protein classes related to immune defense at all stages (Fisher’s test; *p* < 0.05). (**C**) Identity of proteins (or gene ID) present in the PVF during embryonic development and their presence (purple) or absence (yellow) at different stages. The presence of a protein at 0 dpf suggests a maternal origin, since samples were taken before the MBT; the absence of a protein at this stage suggests a zygotic origin, while the presence before and after MBT, suggests a mixed maternal-zygotic origin. The gene list associated with each protein found in the PVF was organized by their protein superfamilies, families, and/or molecular functions. For example, the A2M superfamily corresponds to the “serine protease inhibitors” in Figure 1B. (**D**) Zebrafish PVF can agglutinate live bacterial cells. The images show in vitro agglutination assays using fluorescent *E. coli* and *S. typhimurium*, which cluster in the presence of PVF. (**E**) PVF protects the zebrafish embryo from bacterial infection. Zebrafish embryos challenged at (i) 1 dpf and (ii) 2 dpf. The result reveals an augmented survival rate in the presence of PVF (Kruskal–Wallis test: ****p < 0.0001; *** *p* < 0.001; * *p* < 0.05). (**F**) The three main elements of early embryonic defense against pathogens in fish. The PVF constitutes the central element in a tripartite system of embryonic defenses against environmental pathogens. If an organism, chemical compound, or even a physical element from the exterior succeeds in crossing the chorion, it still must pass through the PVF to reach the embryo.

To carry out proteomics of the PVF, we recovered it from embryos at three different stages of development (Figure S1): 0–2 h post fertilization, hpf (early blastula), 24 hpf (pharyngula), and 48 hpf (pre-hatching). Proteomic analysis of PVF from these developmental timepoints showed, on the one hand, a group of proteins that were stage-specific and, on the other, a group of proteins that remained constitutively present in the PVF from blastula until hatching. The number of protein sequences identified and those ontologies overrepresented at each stage are shown in Figure 1A,B. Interestingly, our results revealed that most of the proteins identified were constitutive (85) and were related to defensive functions, such as the complement system, cytokine activity, and others (Figure 1B). Our results also strongly suggested that these proteins were of maternal origin, since they were present in the 0–2 hpf embryo, before the activation of the embryonic genome that occurred three hours after fertilization, at the mid-blastula transition (MBT) [14].

Proteomic analysis of the PVF reveals that this extraembryonic fluid is rich in defensive proteins related to the innate immune system (Figure 1C). We found numerous lectins (sugar binding proteins) that can act as pattern recognition receptors (PRRs), antifreeze agents, and even venoms [15]. The lectins identified include two isoforms of the C reactive protein (CRP and CRP2), of the pentraxin superfamily, which are well known PRRs present in the circulation of invertebrate and vertebrate animals. These molecules possess the ability to interact with bacteria, fungi, yeast, and other pathogens and parasites acting as agglutinating or opsonizing agents through the activation of the complement system [16,17]. Several C-type lectin sequences with galactose and rhamnose binding domains were identified. Galactose and rhamnose are abundant carbohydrates in the cell wall of prokaryotes with roles in virulence and viability for bacterial pathogens [18,19,20]. We hypothesized that the high number of sugar binding proteins present in the PVF were likely to provide this fluid with opsonization or agglutination activity. In addition, glycolytic enzymes such as the acidic chitinases, Chia.3 and Chia.4, were present in the PVF. Chitin, the substrate of chitinases, is a polysaccharide present in the cell wall of fungi and the exoskeleton of insects, helminths, and crustaceans [21]. These enzymes, with unknown function in the zebrafish embryo, present ubiquitous expression in the zebrafish embryo until 24 hpf, and have been suggested to be important during development [22]. We suggest that PVF chitinases could have a possible antifungal and/or antiparasitary function to protect the early embryo, as other glucosidases found can probably modify the cell surface of other pathogens, preventing their binding to the embryonic epithelia.

We were also intrigued by the presence of Transferrin-a (TFA) in the PVF at 2 dpf. TFA is an iron transporter essential for erythropoiesis, but also very important for the acute phase immune response in animals [23], including zebrafish [24]. TFA can limit the availability of free iron needed by pathogenic bacteria, reducing their survival; this innate defense mechanism is called nutritional immunity [25,26]. While the mRNAs for *tfa* and its receptors, *tfr1a* y *tfr1b*, were detected in previous work at 3,5 hpf [13], indicating maternal expression of these genes, we did not detect the TFA protein in the PVF before the MBT. This suggests that either the *tfa* mRNA was not translated in the early embryo, or that the TFA protein was not present in the PVF until after 1 dpf. Additionally, the reported expression pattern of *tfa* mRNA in the zebrafish yolk syncytial layer [27] suggests that this tissue could be the source of this protein in the PVF at the pre-hatching stage. The mechanism leading to the presence of TFA and other proteins that have not been reported to be secreted in the PVF is unknown. A possible explanation could be the presence of dead epidermal or epithelial cells in the PVF, or that the origin of some of these proteins could be hatching gland cells, which secrete proteases that change the structure of the chorion in pre-hatching stages [28]. However, the exact content of the secretory granules of hatching gland cells is also unknown. In any case, more studies should be carried out to solve this issue.

Interestingly, we found the presence of eight isoforms—originating from six different genes—of the α-2-macroglobulin (a2m) protease inhibitors, at all stages analyzed. The a2m superfamily members are universal serine protease inhibitors with a broad substrate diversity that can neutralize diverse kinds of proteases without peptide bond cleavage using a fly trap molecular mechanism. The a2m is a central element in the innate immunity of invertebrates and vertebrates that has numerous described functions, including the capture and inhibition of virulence factors, such as microbial proteases secreted to facilitate penetration of the host’s barriers [29]. It is interesting to note that a2m has been proposed as the evolutionary ancestor of the C3 protein of the complement system [30,31]. Thus, our proteomic analysis suggests that the zebrafish PVF contained potent defensive molecules that could have had an active role in the protection of the embryo against external threats.

Using bacteria and in vitro experiments, we were unable to detect antibiotic activity in the PVF (not shown). However, considering the high levels of lectins we found in the PVF, we tested its agglutination capacity by assaying it directly on live pathogens using two bacterial strains expressing a GFP reporter. Our results showed a strong agglutination capacity of zebrafish PVF on Gram negative bacterial cells such as *Escherichia coli* and *Salmonella typhimurium* (Figure 1D). This agglutination reaction was strongest when fresh PVF was assayed, and the agglutination activity of the PVF could still be detected with a 10% dilution (not shown). We observed that storage of PVF reduced its agglutination capacity, though activity was retained in a 50% PVF–PBS solution after 2 weeks’ storage at −80 °C. Similar agglutination was also observed in a serological test using the fish pathogen *Edwardsiella tarda* (Figure S3). These results strongly suggest that the sugar binding proteins from the PVF could have been acting as agglutinins, as has been previously described for this group of proteins [32].

Finally, in order to demonstrate that the PVF can protect the developing embryos from infection by bacterial pathogens, we designed a bacterial pathogen challenge experiment that requires small volumes of PVF. Zebrafish embryos (lacking their chorions) were exposed by immersion to the fish pathogen *E. tarda*, in the presence or absence of PVF, and larval survival was monitored for four days post infection (dpi). Our results revealed that PVF by itself can effectively protect zebrafish embryos from bacterial infection (Figure 1E). The survival rate of individuals exposed to the bacterial suspension in PVF was significantly higher than those exposed to the suspension of bacteria in PBS (positive control) and presented no significant difference with zebrafish embryos not exposed to the pathogen (negative control). A previous report [33] has suggested a possible function of PVF against viral infection. In that case, viral infection was tested by bathing 2 dpf embryos without their chorions, or with their chorions pierced, with virus. Although embryos with pierced chorions were more susceptible, the effect of the chorion itself was not tested in this experiment. In contrast, our results clearly demonstrated that zebrafish PVF itself can exert a defensive role against bacterial infections in vivo, protecting the zebrafish embryo from environmental pathogens before the development of the immune system.

Our results provide evidence for an immune defensive role for the PVF, an extraembryonic, maternally inherited fluid that surrounds the zebrafish embryo between fertilization and hatching. We revealed, through proteomic profiling, that the composition of the PVF was complex and changed dynamically during development. A large fraction of the proteins we detected in our assay are known to participate in innate immunity and host defense. In addition, we showed, both in vitro and in vivo, that PVF had antibacterial activity. Our findings further strengthen the concept that the PVF was a crucial component of the protective mechanisms against environmental threats in animals with external development (Figure 1F). The chorion is the first defensive physical barrier for the embryo. It provides passive protection against dehydration, mechanical injury, and acts as a physical barrier against microorganism and parasite entry. However, the chorion is not impenetrable, due to the presence of pores in its structure and its vulnerability to the action of proteases. Between the chorion and the embryo, any environmental threat must cross the perivitelline space that is filled with PVF and its innate immune proteins. Our hypothesis was that the diversity of defensive proteins present in the PVF would strongly reduce the ability of pathogens and other foreign agents to reach and penetrate the embryo, as shown in the graphic model in Figure 1F. Our results established that this fluid was a second defensive barrier, as it actively prevented pathogens from reaching the embryo through agglutination, and likely decreased virulence through the enzymatic modification of their cellular envelopes and/or by blocking the action of bacterial proteases necessary to penetrate the embryonic epithelia. We also suggested that bacteria may be subject to nutritional immunity, through sequestration of available iron by PVF proteins.

Throughout embryogenesis, from fertilization until the maturation of the innate immune system near hatching, the embryonic tissues and the components stored in the yolk comprise the last line of defense against pathogens and other threats that have successfully crossed the chorion and the PVF before reaching the embryo. Later, after hatching and the consumption of all yolk reserves, the embryonic innate immune system will be the main defensive agent against biological threats.

We propose that the zebrafish perivitelline fluid is an inherited extraembryonic defense system against environmental threats, a role that has not been previously described for this structure in any vertebrate animal.

## Supplementary Materials

The following are available online at https://www.mdpi.com/2218-273X/10/9/1274/s1, Figure S1: A summary of the workflow to establish the defensive function of the PVF. Figure S2: PVF location in the embryo and isolation technique. Figure S3: PVF agglutinates bacteria.
