# Supplementary material for: The Zebrafish Perivitelline Fluid Provides Maternally-Inherited Defensive Immunity
Source: Biomolecules. 2020 Sep 3;10(9):1274. doi: 10.3390/biom10091274 (PMC7564447; doi:10.3390/biom10091274)
Supplement: Supplementary file 1 [file biomolecules-10-01274-s001.pdf]

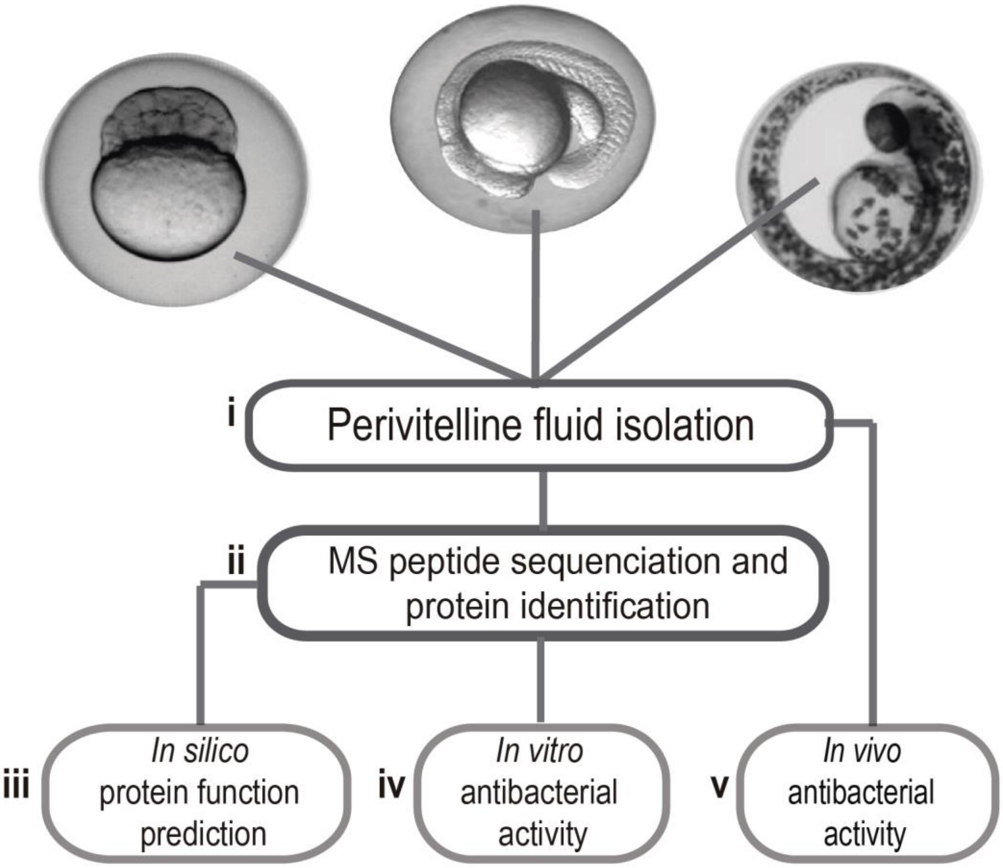

Figure S1. A summary of the workflow to establish the defensive function of the PVF. (i) *PVF isolation* from early blastula stage (0 to 2 hpf, before the mid blastula transition, MBT, occurs), 1 dpf and 2 dpf embryos before hatching. (ii) PVF samples were collected and processed for sequencing by MS/MS. (iii) The proteomic analysis made it possible to predict protein functions. Finally the (iv) *in vitro* and (v) *in vivo* PVF testing, were done in order to demonstrate the antimicrobial properties of the PVF.

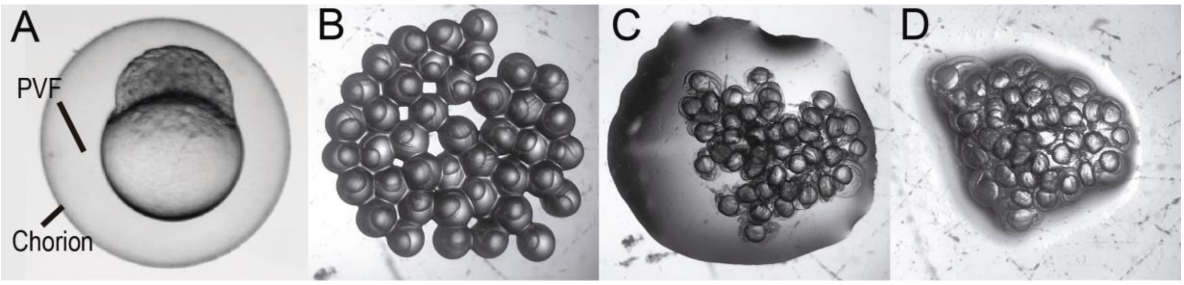

Figure S2. PVF location in the embryo and isolation technique. (a) A zebrafish blastula shows the perivitelline space that contains the PVF where the embryo develops. To isolate the PVF, (b) embryos in their chorions are placed in a clean and smooth glass surface and all the remaining liquid is aspirated, then (c) the chorion is mechanically broken to release the PVF, (d) which is collected with a micropipette and placed in a centrifuge tube on ice and stored at -80ºC for no longer than two weeks before functional analysis.

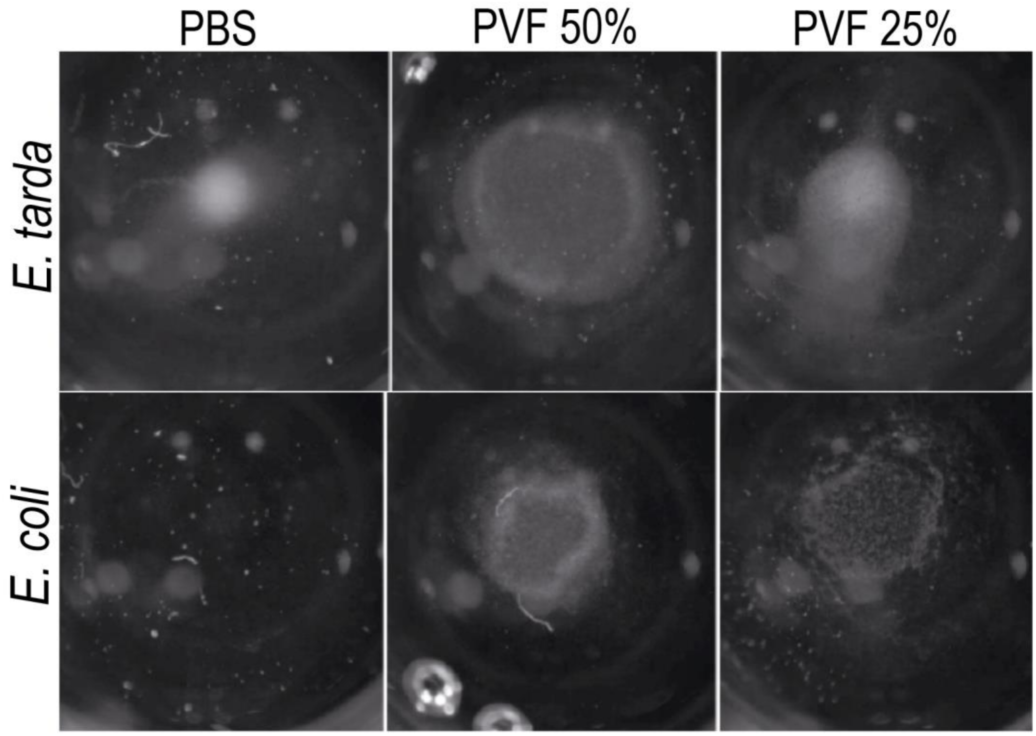

Figure S3. PVF agglutinates bacteria. The serological test in multiwell plates confirms the agglutination reaction observed by fluorescent microscopy with *E. coli*; this reaction also occurs with *E. tarda* cells in the presence of diluted PVF. Images were taken after four hours of incubation at 28ºC. The formation of a bacterial mat on the bottom of the well is an indicator of a positive agglutination reaction.
